# High-Level Expression, Purification and Characterization of A Recombinant *Plasmodium vivax* Apical Membrane Antigen 1: Implication for vivax Malaria Vaccine Development

**DOI:** 10.22074/cellj.2015.12

**Published:** 2015-10-07

**Authors:** Maryam Salavatifar, Sedigheh Zakeri, Nasim Hayati Roodbari, Navid Dinparast Djadid

**Affiliations:** 1 Department of Biology, Science and Research Branch, Islamic Azad University, Tehran, Iran; 2 Malaria and Vector Research Group (MVRG), Biotechnology Research Center (BCR), Pasteur Institute of Iran, Tehran, Iran

**Keywords:** Malaria, *Plasmodium vivax*, Apical Membrane Antigen-1, Vaccine

## Abstract

**Objective:**

The apical membrane antigen-1 (AMA-1) is considered as a promising candidate for development of a malaria vaccine against Plasmodium parasites. The correct conformation of this protein appears to be necessary for the stimulation of parasite-inhibitory responses, and these responses, in turn, seem to be antibody-mediated. Therefore, in the present investigation, we expressed the *Plasmodium vivax* AMA-1 (PvAMA-1) ectodomain in *Escherichia coli (E. coli)*, purified it using standard procedures and characterized it to determine its biological activities for it to be used as a potential target for developing a protective and safe vivax malaria vaccine.

**Materials and Methods:**

In this experimental investigation, the ectodomain of PvAMA-1 antigen (GenBank accession no. JX624741) was expressed in the *E. coli* M15pQE30 expression system and purified with immobilized-metal affinity chromatography. The correct conformation of the recombinant protein was evaluated by Western blotting and indirect immunofluorescence antibody (IFA) test. In addition, the immunogenic properties of PvAMA-1 were evaluated in BALB/c mice with the purified protein emulsified in Freund’s adjuvant.

**Results:**

In the present study, the PvAMA-1 ectodomain was expressed at a high-level (65 mg/L) using a bacterial system. Reduced and non-reduced sodium dodecyl sulfate-polyacrylamide gel electrophoresis (SDS-PAGE) as well as Western blot analysis confirmed the appropriate conformation and folding of PvAMA-1. The evaluation of immunogenic properties of PvAMA-1 showed that both T helper-1 and 2 cells (Th1 and Th2) responses were present in mice after three immunizations and persisted up to one year after the first immunization. Moreover, the antibodies raised against the recombinant PvAMA-1 in injected mice could recognize the native protein localized on *P. vivax* parasites.

**Conclusion:**

We demonstrate that our recombinant protein had proper conformation and folding. Also, there were common epitopes in the recombinant forms corresponding to native proteins. These results; therefore, indicate that the expressed PvAMA-1 has the potential to be used as a vivax malaria vaccine.

## Introduction

*Plasmodium vivax (P. vivax)* is the second most common prevalent species and broadly distributed human parasite that reasons malaria morbidity among people of all ages in Africa, the Middle East, Asia and Latin America (1,2). Although *P. vivax* has less mortality than Plasmodium falciparum, it leads to a relapsing and disabling disease, and has an enormous socioeconomic impact on the community (3,5). In recent years, re-emergence of *P. vivax* in eradicated areas, the emergence of drug-resistant strains (6), and severe disease and death (7,8) have been indications of public health importance of the *P. vivax* parasite which could be a major obstacle to malaria control and elimination programs. Considering the World Health Organization (WHO) malaria elimination and eradication programs, it has been accepted that eradication is not possible with the current tools. Therefore, the research and development of new drugs, diagnostic tests, insecticides and a cost-effective deployable vaccine will be needed to facilitate eradication. However, one of the problems in developing a malaria vaccine is the large-scale production of the target protein with the correct conformation (9). 

Several antigens have been already identified that have the potential to be used in subunit vaccine development against *P. vivax* (10,15). Some of these antigens, expressed at different stages of the parasite life cycle, are the merozoite surface protein, Duffy-binding protein and apical membrane antigen-1 (AMA^1^) (16,18). Among them, AMA-1 is one of the most promising vaccine candidates against the malaria parasite (19). This antigen is expressed in *P. vivax* at two critical stages of the parasite life cycle, sporozoite and merozoite (20,21). In the sporozoite stage, AMA-1 is critical for hepatocyte invasion and in merozoite stage, it is essential for red blood cell invasion (22). This antigen also belongs to the type I integral membrane protein with a small C-terminal cytoplasmic domain, a single transmembrane region as well as an N-terminal ectoplasmic region (23). AMA-1 has 556 to 563 amino acids in most Plasmodium species (24). Its cytosolic region consists of 50 amino acids while its ectodomain consists of 16 invariant cysteine residues. These cysteines form eight disulfide bonds, which divide the protein into three distinct domains (i.e. DI, DII, and DIII) (25). Using X-ray crystallography and/or nuclear magnetic resonance (NMR), structural studies on *P. vivax* AMA-1 (PvAMA-1) have shown that DI and DII are structurally similar to each other and belong to the PAN module ( commonly found in proteins with various adhesion functions (22,24,26,27). 

In developing a subunit vaccine, proper folding and conformation of the target protein are essential for inducing an effective immune response (28). Accordingly, immunogenicity of an AMA-1-based vaccine depends critically on its possible conformational epitopes (26). Furthermore, PvAMA-1 contains three possible N-glycosylation sites, however, in the native form of this protein, all of these three sites are nonglycosylated (29). Therefore, these glycosylations may affect the immunogenicity of the PvAMA-1 protein. 

Earlier studies have demonstrated that immune responses elicited by the full ectodomain of PvAMA-1 or its DII in mice induce high levels of IgG1 antibodies, followed by IgG2a, IgG2b and lower levels of IgG3 (30,31). These antibodies react to appropriate conformational epitopes stabilized by disulfide bonds since immunization with the reduced and alkylated AMA-1 fails to protect mice against the challenge with *Plasmodium chabaudi* (32). In addition, several studies on naturally acquired immunity to PvAMA-1 have illustrated that cytophilic IgG1 and IgG3 antibodies against this protein are related to protection (33). Moreover, other investigations regarding the naturally acquired human immune response to PvAMA-1 undertaken in different areas of Brazil and Sri Lanka (33,35) emphasized that AMA-1 is extremely immunogenic during human malaria infection. 

*P. vivax* is the most prevalent malaria species in Iran representing more than 88% of the clinical cases reported annually (Iranian Health Ministry, unpublished, 2013). Moreover, as *P. vivax* accounts for most of the malaria cases in Iran and also its neighboring countries (Afghanistan and Pakistan), it is essential to develop new intervention tools (such as vaccine) against this challenging species. Therefore, in the current study, we expressed PvAMA-1 in *Escherichia coli (E. coli)*, purified it with standard procedures and characterized the expressed protein, thus demonstrating to evaluate its potential for developing protective and safe vivax malaria vaccine. 

## Materials and Methods

### Cloning PvAMA-1 into pGEM-T easy vector

In this experimental study, to express the recombinant PvAMA-1 (rPvAMA-1), genomic High-Level Expression of Recombinant PvAMA-1 DNA obtained from Iranian individuals with patent *P. vivax* infection (Chabahar, Sistan and Baluchistan Province, South-East Iran) and the known sequences of PvAMA-1 ectodomain (GenBank accession no. JX624741, amino acids 42 to 487) were amplified as described earlier (36). Briefly, the polymerase chain reaction (PCR) cycling conditions were 95˚C for 5 minutes, followed by 30 cycles of 60˚C for 1 minute, 72˚C for 1 minute and 94˚C for 1 minute with a final extension at 72˚C for 30 minutes. The primers were designed in Malaria and Vector Research Group (MVRG) using Gene Runner software based on the sequence of *P. vivax Sal-1 ama-1* gene (accession no. AF063138) from nucleotides 124 to 1461. In order to use these primers for subcloning AMA-1 into the pQE30 plasmid, *BamHI* and *SmaI* restriction sites were designed in oligonucleotide primers as follow: 

AMAF: 5´-ATTATGGATCCGGGCCTACCGTTGAGAG-3΄ (*BamHI* site at nucleotides 124-140 and underlined) 

AMAR: 5´-TTCACCCGGGTTATAGTAGCATCTGCTTG-3´ (*SmaI* site at nucleotides 1446-1461 with the stop codon in italics). 

PCR product was purified from agarose gel using a DNA Extraction Kit (Qiagen, Germany) and inserted into the pGEM-T easy vector (Promega, Madison, WI, USA). The ligation mixtures were transformed into competent *E. coil* DH5α cells, and the transformed clones were selected on the LuriaBertani medium containing 100 μg/ml ampicillin, 0.2 mM isopropyl-β-D-thiogalactopyranoside (IPTG) and 0.04% X-gal. The clones were then confirmed by plasmid extraction followed by EcoRI digestion. For final confirmation, the recombinant plasmid was sequenced using AMAF and AMAR primers. Each sequence was aligned with the sequence of *Sal-1 ama-1* gene (Accession no. AF063138) using ClustalW (http://www.ebi.ac.uk/Tools/msa/clustalw2/). 

### Subcloning PvAMA-1 into pQE30 expression vector

To subclone PvAMA-1 into the pQE30 vector, we removed the fragment corresponding to the PvAMA-1 sequence from the pGEM-T easy PvAMA-1 vector using *BamHI* and *SmaI* restriction enzymes. The fragments were then ligated to the *BamHI* and *SmaI* sites of pQE30 (Qiagen, Germany) making the hexahistidine-tag (His-Tag) available in the N-terminus of PvAMA-1 to facilitate further purification. 

### Expression of PvAMA-1 in E. coli M15 (pREP4)

rPvAMA-1 was expressed in the *E. coli* M15 (pREP4) expression system (Qiagen, Germany). Briefly, a single positive clone was considered for AMA-1 expression and expanded in Terrific Broth (TB) and Luria-Bertani media (2×), containing ampicillin (100 μg/ml) and kanamycin (25 μg/ml) while being shaked (150 rpm) at 30˚C and 37˚C until OD_600nm_reached 0.6-0.8. The expression of PvAMA-1 was induced with 0.1, 0.2, 0.5, and 1 mM IPTG (Sigma, USA) to optimize the expression conditions. The culture was then further grown with shaking. The *E. coli* cells were harvested by centrifugation for 1, 2, 3, and 4 hours after induction and analyzed by 12% sodium dodecyl sulfatepolyacrylamide gel electrophoresis (SDS-PAGE) gel under reducing conditions. The highest expression was obtained in TB medium containing 0.2 mM IPTG at 37˚C for 4 hours after induction. The expression level of rPvAMA-1 was measured by a densitometer (BioRad, USA). Bovine serum albumin (BSA, Sigma, USA) was used as a standard to set up a standard curve from which the unknown protein concentration could be determined. Also, a 1 mg/ml BSA stock solution was used to prepare a standard two-fold dilution series (1,000, 500, 250, 125, and 62.5 mg/L). 

### Purification of rPvAMA-1

To purify the His-Tag fused rPvAMA-1 using Ni ^2+^-nitrilotriacetic acid agarose resin (Ni-NTA agarose, Qiagen, Germany), we applied immobilized-metal affinity chromatography under denaturing conditions. Briefly, the cells containing the inclusion bodies of rPvAMA-1 were resuspended in a lysis buffer (8 M urea, 20 mM Tris-HCl, 30 mM imidazole and 1 M NaCl, pH=7.9) and incubated at 4˚C for 1.5 hours. The cells were then lysed with 10 sonication cycles (Ultraschallprozessor, Germany) each consisting of 20-second pulses with 40-second intervals. The bacterial lysate was centrifuged at 8,000 rpm at 4˚C for 10 minutes. The Ni-NTA agarose was equilibrated by an equilibration solution containing 8 M urea, 20 mM TrisHCl, 40 mM imidazole and 1 M NaCl, pH=7.9. Afterward, the supernatant of the bacterial lysate containing the recombinant protein was incubated with equilibrated Ni-NTA agarose at 4˚C for 2 hours. The resin was then packed into a column and washed with a 10-column volume of wash buffer (6 M urea, 20 mM Tris-HCl, 60 mM imidazole and 1 M NaCl, pH=7.9). The bound protein was then eluted with an elution buffer containing 4 M urea, 20 mM Tris-HCl, 300 mM NaCl and 200 mM imidazole, pH=7.9. All elutes were analyzed by 12% SDS-PAGE gel under reducing and non-reducing (in the absence of 2 ME and boiling) conditions. Then, the elutes containing PvAMA-1 were desalted with Econo-Pac 10DG columns (BioRad, USA) according to the manufacturer’s instructions and concentrated with a concentrator (Eppendorf, Germany). The concentration of the purified protein was determined by Bradford’s assay at 595 nm (37). 

### Western blotting analysis

To determine the expression of PvAMA-1 and confirm the presence of the isolated proteins, we performed immunodetection. After separating by SDSPAGE gel, the proteins were transferred to a nitrocellulose membrane at 18 v for 1 hour (Trans-Blot Semi-Dry, BioRad, USA). Then, the membrane was blocked in 20 ml blocking buffer (2% BSA) at 4˚C overnight and washed three times with wash buffer [0.05% Tween-20 in phosphate-buffered saline (PBST, 1×)] for 20 minutes. Next, we incubated the membrane with *P. vivax*-infected human sera (1:100 dilution) and/or anti-penta-His antibody (1:1,500; Qiagen, Germany) in both reduced and non-reduced conditions on a shaking platform at room temperature (RT) for 2 hours. The membrane was washed again three times with wash buffer (PBS-T, 1×) for 20 minutes. It was then incubated for 1.5 hours with anti-human IgG antibody peroxidase (1:6,000) for human sera and/or with anti-mouse IgG antibody peroxidase for anti-His antibody (1:2,000) as secondary antibodies for 1.5 hours. The membrane was further washed three times as described above. The reaction was developed by an enzyme-specific substrate, 3, 3΄-diaminobenzidine (DAB, Sigma, USA), and the reaction was stopped by 2 N sulfuric acid. The serum samples were obtained from the residents of Tehran Province with no pervious exposure to malaria (outside malaria-endemic areas) as negative controls. 

### Endotoxin testing

Cell wall endotoxins of Gram-negative bacteria are potent pro-inflammatory compounds that have been shown to cause both acute and chronic diseases. Endotoxin injection can cause acute chills, fever, organ failure, and death (38). Thus, most drugs and biological materials are needed to with stand rigorous testing to ensure that they contain no more than a specified quantity of endotoxin (38). The level of bacterial endotoxin was determined using the LAL chromogenic kit (Lonza, USA) at the Quality Control Unit of the Recombinant Protein Production Complex of Pasteur Institute of Iran. 

### Mice immunization

To produce polyclonal antibodies to rPvAMA-1, inbred female BALB⁄c mice (6-8 weeks old) were obtained from the Department of Laboratory Animal Science at Pasteur Institute of Iran (Tehran) and immunized with the purified recombinant protein. A group of mice (n=10) was immunized subcutaneously at the base of the tail with rPvAMA-1. For priming, the mice received 100 μl (40 μg⁄mouse) rPvAMA-1 emulsified in complete Freund’s adjuvant in 1:1 ratio (Sigma, USA). However, the mice in the first control group were immunized with PBS in complete Freund’s adjuvant and the second control group were immunized with PBS alone. The endotoxin concentration of the purified PvAMA-1 was 1.56 EU/ml. Therefore, the injection of 40 µg rPvAMA-1/mouse was deemed as an acceptable amount (0.0624 EU) of endotoxin introduced into each mouse. All mice were boosted at two and four weeks after the first immunization with the same protocol but with incomplete Freund’s adjuvant (Sigma, USA). Two weeks after the last immunization (six weeks after the first immunization), the mice were bled, and the sera were kept at -20˚C until use. To assess antibody persistence, antibody levels in mouse sera were monitored at 30 and 56 weeks after the first immunization. All of the experimental protocols were approved by the Committee of Animal Ethics of the Pasteur Institute of Iran. 

### Evaluation of antibody responses by the enzyme linked immunosorbent assay (ELISA)

Immunized mouse sera were evaluated for antiPvAMA-1-specific antibodies by ELISA. Briefly, Maxisorp flat-bottom 96-well microplates (Greiner Labortechnik, Nurtingen, Germany) were coated with 250 ng of purified rPvAMA-1 in 0.06 M carbonate-bicarbonate buffer (pH=9.6) and incubated at 4˚C overnight. After washing with PBS 1× containing PBS-T, the plates were blocked with 200 µl PBS 1×, containing 2.5% BSA (pH=7.4) at RT for 1.5 hours. After washing step, the immunized mouse sera (diluted 1:200 in PBS-T containing 0.5% BSA) were added to each well. The plates were then washed and incubated with 100 µl goat antimouse IgG horseradish peroxidase (diluted 1:25,000 in PBS, Sigma, USA) at RT for 1 hour. An enzyme-specific substrate [3, 3΄, 5, 5΄-Tetramethylbenzidine (TMB), Jahan Alcohol Teb, Arak, Iran] was subsequently added to each well. The ELISA cut-offs were obtained from the average of the negative sera (n=10, normal mouse sera) plus 3 standard deviations (SD). In order to determine the IgG subclass, instead of goat anti-mouse IgG horseradish peroxidase, 100 µl of either goat anti-mouse IgG1, IgG2a, IgG2b, or IgG3 (diluted 1:1,000 in PBS, Sigma, USA) were added to each well and incubated at RT for 1 hour. The plates were then incubated with 1:10,000 dilution of anti-goat IgG horseradish peroxidase (Sigma, USA) at RT for 1 hour and developed by TMB. The reaction was stopped by 2 N sulfuric acid and absorbance was measured at 450 nm. 

### Indirect immunofluorescence antibody (IFA) test

In this investigation, indirect immunofluorescence antibody (IFA) test was performed to test the ability of anti-PvAMA-1 sera of the immunized mice in recognizing the native form of PvAMA-1 antigen on merozoite surface as well as to determine the extent of similarity between epitopes in recombinant forms and corresponding native proteins. For this reason, multispot parasite slides were prepared from *P. vivax*infected patients, air-dried and then fixed with cold acetone for 10 minutes. Diluted polyclonal mouse sera (1:10-1:51, 200) in PBS-T were then added to spots and incubated in a wet chamber for 30 minutes. After washing three times with PBS (pH=7.4), each well was covered with 25 μl fluorescein-conjugated anti-mouse or antihuman (in case of a positive control) polyvalent IgG (1:40) and then left in a wet chamber for 30 minutes. After repeating the washing process, coverslips were placed on each slide and examined under a fluorescence microscope (Nikon E200, Tokyo, Japan) with a 100× oil immersion objective. 

### Statistical analysis and ethical considerations

The differences in the amount of antibody responses in different groups were analyzed by oneway ANOVA. The differences in the level of antibody responses at 6, 30, and 56 weeks after the first immunization were analyzed by paired sample t test. A P value <0.05 was considered statistically significant. 

## Results

### Construction, expression and purification of rPvAMA-1 in *E. coli*

**Fig.1:**
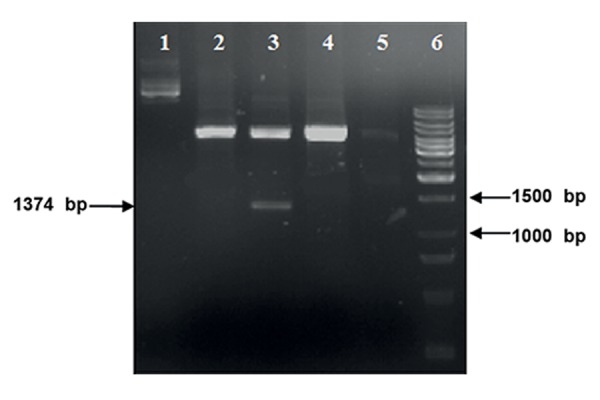
The confirmation of PvAMA-1 cloning in pQE30 plasmid using digestion with *BamHI* and *SmaI* restriction enzymes and electrophoresis on a 1% agarose gel. Lane 1; Uncut pQE30, lanes 2, 4, and 5; pQE30 without PvAMA-1 (negative clones), lane 3; Recombinant plasmid with insert (pQE30- PvAMA-1) and lane 6; 1 kb molecular weight (Fermentas, USA).

We successfully amplified the ectodomain of *pvama-1* gene (1374 bp), cloned it into the pGEMT easy vector and subcloned it into the pQE30 plasmid. The digestion of recombinant pQE30- PvAMA-1 with *BamHI* and *SmaI* restriction enzymes showed a similar band size on agarose gel verifying this recombinant plasmid (Fig .1).

The selected variant form was cloned and expressed in *E. coli* M15 strain and fused to the His-Tag using the pQE30 vector (Qiagen, Hilden, Germany). The optimum condition for PvAMA-1 expression was obtained in TB medium and 0.2 mM IPTG was added to culture when OD_600nm_ reached 0.6-0.8. The expression level of rPvAMA- 1, measured with a densitometer, was 65 mg/L. The analysis of the purified rPvAMA-1 by SDS-PAGE showed a molecular weight of 52 kDa, which was in good agreement with the expected molecular mass of 52 kDa (Figes.2, 3). Altogether, these results confirmed the purity and fidelity of rPvAMA-1. Furthermore, reduced and nonreduced SDS-PAGE (Fig .4A) as well as Western blot analysis (Fig .4B) confirmed correct conformation and folding of this protein.

**Fig.2:**
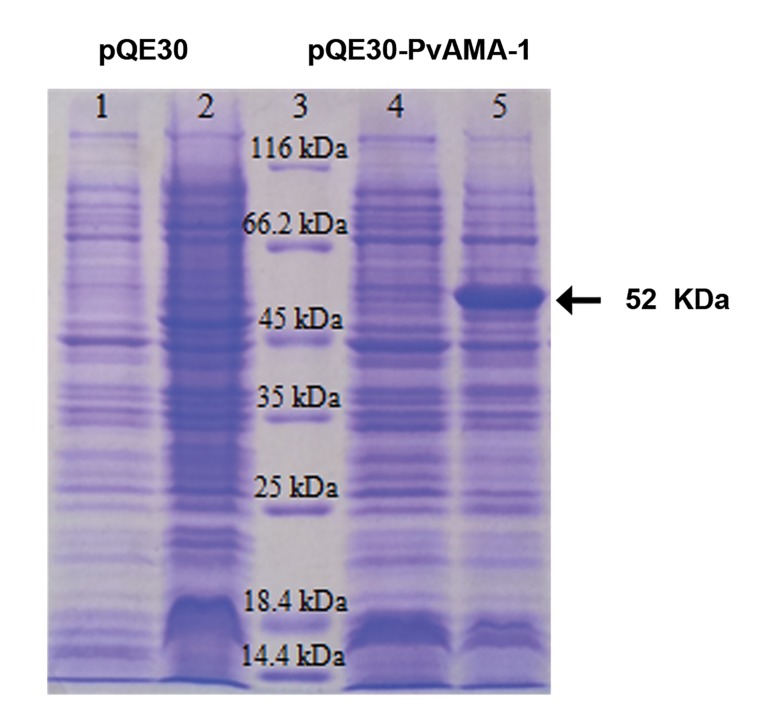
Sodium dodecyl sulfate-polyacrylamide gel electrophoresis (SDS-PAGE) analysis of PvAMA-1 expressed in the *E. coli* M15-pQE30 system. Lane 1; The pellet of E. coli M15-pQE30 before induction, lane 2; The pellet of E. coli M15-pQE30 4 hours after induction, lane 3; Protein size marker (14.4-116 kDa, Fermentas, USA), lane 4; The pellet of E. coli M15-pQE30-PvAMA-1 before induction and lane 5; The pellet of *E. coli* M15-pQE30- PvAMA-1 4 hours after induction.

**Fig.3:**
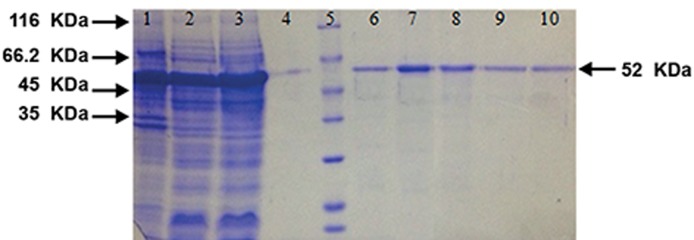
Sodium dodecyl sulfate-polyacrylamide gel electrophoresis (SDS-PAGE) analysis of PvAMA-1 purification using metal affinity chromatography. Lane 1; Bacterial pellet after sonication and centrifugation, lane 2; Bacterial supernatant after sonication and centrifugation, lane 3; Unbounded proteins passed from column (flow-through), lane 4; Washed solution after passing from column, lane 5; Size marker (14.4-116 kDa, Fermentas, USA) and lanes 6-10; Elutions 1 to 5 (purified PvAMA-1).

**Fig.4:**
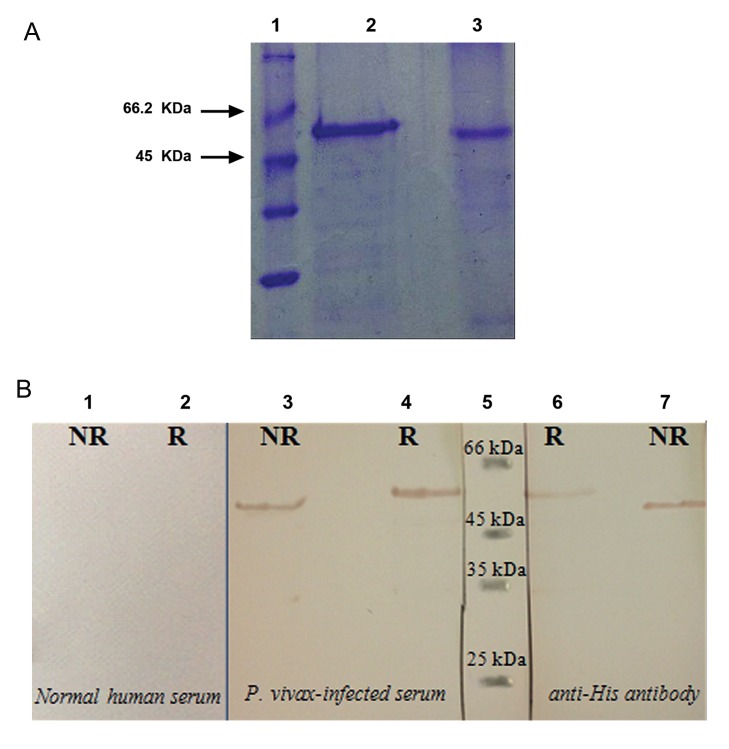
Analysis of reduced and non-reduced rPvAMA-1. A. Sodium dodecyl sulfate-polyacrylamide gel electrophoresis (SDS-PAGE) analysis of purified PvAMA-1 ectodomain. Lane 1; Size marker (14.4-116 kDa, Fermentas, USA), lane 2; Reduced form and lane 3; Non-reduced form and B. The confirmation of rPvAMA-1 expression in both reduced and non-reduced forms using sera from *P. vivax*-infected patients and anti-His antibody. Lanes 1 and 2; Normal human serum, lanes 3 and 4; *P. vivax*-infected human serum, lane 5; Size marker (14.4-116 kDa, Fermentas, USA) and lanes 6 and 7; Anti-His antibody. R; Reduced form of rPvAMA-1 and NR; Non-reduced form of rPvAMA-1.

### Humoral immune responses in the immunized mice

**Fig.5:**
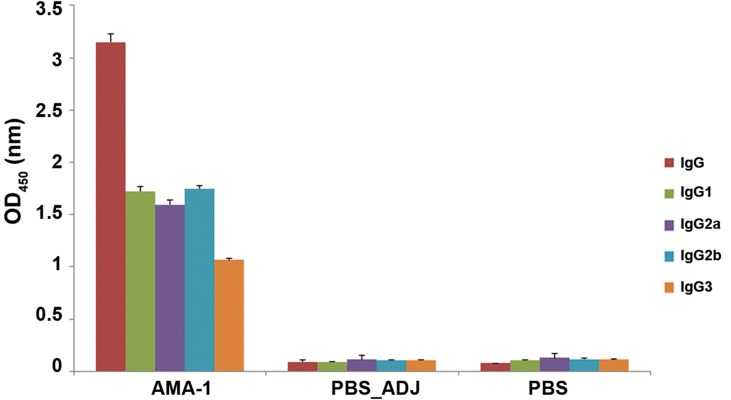
Analysis of IgG and IgG subclass antibodies to the recombinant apical membrane antigen-1 (rPvAMA-1) at six weeks after the first immunization using enzyme linked immunosorbent assay (ELISA). The bars show mean Optical density at 450 nm (OD_450_) of antibodies of pooled mouse sera in each group (at 1:200 dilution), and error bars represent standard deviation (SD). The ELISA cut-offs were obtained from the average of negative sera (n=10 normal mouse sera) plus 3 SD. The cut-off values (OD_450_) for IgG, IgG1, IgG2a, IgG2b and IgG3 were 0.48, 0.56, 0.44, 0.43 and 0.39 for the PvAMA-1 antigen respectively. ADJ; Adjuvant and PBS; Phosphate-buffered saline.

**Fig.6:**
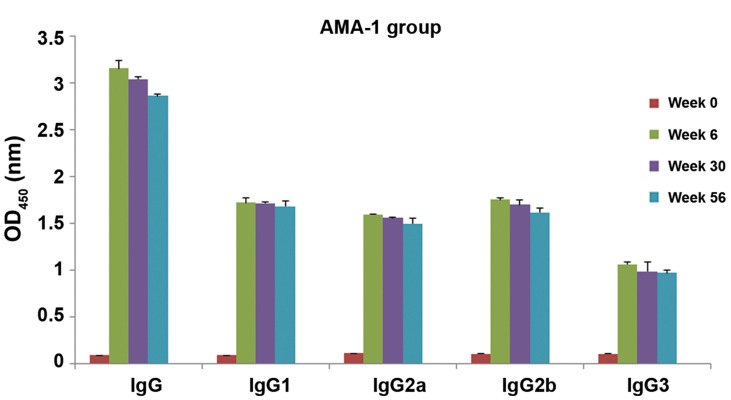
Assessment of persistence of PvAMA-1 IgG, IgG1, IgG2a, IgG2b, and IgG3 antibodies at week 0, 6, 30 and 56 after the first immunization. The bars show mean Optical density at 450 nm (OD_450_) of antibodies of mouse sera, and error bars represent standard deviation.

To characterize humoral immune responses to rPvAMA-1 in immunized mice, we assessed antibody responses using ELISA. The result showed that six weeks after the first immunization, mice immunized with rPvAMA-1 induced high levels of IgG-PvAMA-1 antibodies (mean OD_450_=3.15, cutoff value OD_450_=0.4, Fig .5). However, in comparison with non-immunized mice, no detectable IgGPvAMA-1 was recognized in the control groups (P <0.05, one-way ANOVA, Fig .5). Interestingly, the levels of IgG1, IgG2a, IgG2b, and IgG3 antibodies were 1.72, 1.596, 1.75, and 1.064 respectively at six weeks after the first immunization (Fig .6).

To show persistence of developed IgG and its subclasses in immunized mice, their sera were collected at 6, 30, and 56 weeks after the first immunization. The levels of IgG, IgG1, IgG2a, IgG2b and IgG3 antibodies were 3.04, 1.713, 1.555, 1.7 and 0.985 respectively at week 30 and 2.86, 1.68, 1.495, 1.614 and 0.97 respectively at week 56. The results revealed that the levels of anti-PvAMA- 1 IgG, IgG1, IgG2a, IgG2b, and IgG3 antibodies were significantly increased at six weeks after the first immunization and persisted up to 56 weeks after the first immunization (P>0.05, paired sample t test, Fig .6).

The percentage of antibody level reduction at week 30 after the first immunization was 4% (IgG), 0.6% (IgG1), 2.6% (IgG2a), 3% (IgG2b) and 7.4% (IgG3) while at week 56 after the first immunization, it was 9% (IgG), 2.5% (IgG1), 6.4% (IgG2a), 8% (IgG2b) and 9% (IgG3).

### Recognition of native AMA-1 on P. vivax parasites by mouse polyclonal antibodies to rPvAMA-1

**Fig.7:**
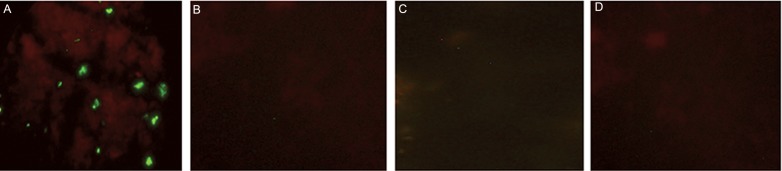
Indirect immunofluorescence antibody (IFA) test for recognition of native form of PvAMA-1 on the P. vivax parasites with polyclonal antibodies induced in mice. Green fluorescence is visible when surface PvAMA-1 on P. vivax are recognized by sera of the immunized mice. A. rPvAMA-1+ CFA⁄ICFA (n=10), B. PBS+CFA⁄ICFA (n=10), C. PBS (n=10) and D. normal mice sera (n=10). CFA; Complete freund’s adjuvant PBS; Phosphate-buffered saline and ICFA; Incomplete freund’s adjuvant.

Anti-rPvAMA-1 antibody produced against the recombinant protein in mice recognized the native protein present on the surface of *P. vivax* merozoite at the late schizont stage with high intensity, as indicated by the grape-like fluorescence pattern (Fig .7A). Consistenty, none of the control mouse sera recognized the native protein on *P. vivax* parasite (Fig .7B-D), confirming that there are common epitopes in recombinant forms corresponding to native proteins. This result further confirms that this recombinant protein had correct conformation and folding.

## Discussion

Malaria elimination and eradication are the final goals of WHO. However, current tools and treatments are insufficient to achieve these goals. As a consequence, development of an effective vaccine is very urgent (39). There are several Plasmodium antigens that are candidates for vaccine development. Among these antigens, AMA-1 is one of the most promising candidates for a malaria blood-stage vaccine. Since an *in vitro* culture system for *P. vivax* is not available, the production of recombinant antigens of this parasite is necessary for development of a vivax malaria vaccine (40). Previous studies have shown that the AMA-1 monoclonal antibodies, which are capable of inhibiting parasite erythrocyte invasion *in vitro*, only recognize disulfide bond-dependent comformational epitopes on the PvAMA-1 antigen (28, 32, 41-43). Therefore, the correct folding of the recombinant protein is critical for vaccine development.

In the present study, we demonstrated that in the *E. coli* expression system, a high amount of PvAMA- 1 with correct conformation could be produced resulting in long lasting humoral immune responses in immunized mice. Moreover, the ectodomain of PvAMA-1 antigen was expressed in the *E. coli* M15-pQE30 expression system with a yield of 65 mg/L. This yield was 30% higher than its expression in the *Pichia pastoris* (GS115) system with a yield of 50 mg/L (44). In addition, PvAMA- 1 was not glycosylated in the native form, however, the expression of this protein in yeast led to glycosylation. Therefore, Kocken et al. (44) performed site-directed mutagenesis using the pAlter II kit, and then the recombinant protein was deglycosylated by N-glycosidase F. In contrast, we expressed this recombinant protein at a high-level without the need to prevent glycosylation and its costly process.

In production of any recombinant protein, peptide affinity tags become very important for purifying recombinant proteins (45). These tags can provide hundredto even thousand-fold target protein purification from raw extracts without any step to remove other cellular materials. Lichty et al. (46) compared the efficiency of eight affinity tags for recombinant protein purification including glutathione S-transferase, maltose-binding protein, hexahistidine, calmodulin-binding peptide, covalent yet dissociable, Strep II, FLAG and heavy chain of protein C. Other similar studies reported that His-Tag provides good yields of tagged proteins from inexpensive, high capacity resins with mild purity from *E. coli* extracts (46). Furthermore, some of the mentioned fusion tags may interfere in immunological assays, while the His-Tag, as the smallest fusion tag, is not immunogenic and is the best fusion tag for immunological assays and vaccine antigens (47). In this regard, in the present study, almost the full-length ectodomain of PvAMA-1 was purified using the His-Tag fusion protein. In previous investigations, the partial fragments of PvAMA-1 were expressed using pGEX-4T1 (48) and the full length of ectodomain of PvAMA-1 was expressed as Trx fusion protein (49). By using such tags, the recombinant protein should be suitable for diagnostic test purposes, however, for immunological assays, the fusion tags must be cleaved. Therefore, adding one more purification step will be costly. Rodrigues et al. (50) used pHIS plasmid to express the ectodomain of PvAMA-1 as a His-Tag fusion protein, but this system is not commercially available.

An additional issue that needs to be considered in the expression of any recombinant protein for vaccine development is its correct folding, especially for rPvAMA-1 production. Previous studies have shown that protective immune responses directed to epitopes are dependent on ectodomain disulfide bounds of PvAMA-1 (17, 28, 32, 42, 51, 52). IFA test confirmed that there are common epitopes in recombinant forms corresponding to the native proteins and bacterial rPvAMA-1 has been correctly folded in a native-like form.

Bouillet et al. (53) showed that mice immunization with rPvAMA-1 produced more IgG1 than Ig- G2a, but after 14 weeks of the first immunization, the antibody level was decreased. This result was also reported by others that IgG1 subclass was produced more than other subclasses using Montanide ISA720 as an adjuvant in immunization of mice (54). Interestingly, in this investigation, using rPvAMA- 1 protein for mice immunization, IgG antibody production revealed a relatively equal level of IgG2b and IgG1 with a substantial amount of IgG2a and a lower amount of IgG3. Therefore, both Th1 and Th2 responses were present in mice after three immunizations and persisted up to one year after the first immunization. The production of IgG1 along with IgG2b is noteworthy because in malaria, pathogen neutralization and clearance are influenced by antibody isotypes. In addition, it is widely accepted that cytophilic antibodies (such as mouse IgG2a) are involved in protective immunity against Plasmodium AMA-1 and other bloodstage antigens (35, 55).

Finally, the expression system we used is simple and cheap and does not need complicated and expensive procedures of genetic engineering. It also produces the recombinant protein with a His-Tag fusion, thus removing any possible interference in immunological assays.

## Conclusion

We show that the expression of PvAMA-1 can be obtained in a prokaryotic expression system with a yield of ~65 mg/L. This expression system is simple, cheap, and does not need complex and expensive manners of genetic engineering. This plasmid expresses the recombinant protein as His- Tag fusion that it does not interfere in immunological assays. Furthermore, the results showed that the antibodies raised against recombinant AMA-1 could recognize the native PvAMA-1 antigen on P. vivax parasites. This observation signifies the suitability of this recombinant protein in serological studies and vaccine development. However, elucidating their function in protection needs additional research.
